# A case report of blunt gastric perforation treated with endoscopic clip closure

**DOI:** 10.1097/MD.0000000000006774

**Published:** 2017-05-05

**Authors:** Ke Li, Xuelian Sun, Guoxing Wang

**Affiliations:** Department of Emergency Capital Medical University Affiliated Beijing Friendship Hospital, Beijing, China.

**Keywords:** computed tomography, gastric perforation, gastroscopy

## Abstract

**Rationale:**

Gastroscopymay not only allow identification of gastric injury, but may also facilitate prompt repair.

**Patient concerns:**

A 39-year-old male patient was admitted 3 hours after abdominal injury caused by penetration of a screwdriver. Physical examination and computed tomography showed no evidence of gastric injury. However, 3000 mL fresh blood was vomited during the subsequent observation period.

**Diagnoses:**

Gastroscopy identified a ruptured artery and gastric perforation.

**Interventions:**

Repaired immediately using titanium clips.

**Outcomes:**

After operation, the patient recovered well without complications. No more bleeding was observed after the operation.

**Lesson subsections asper style:**

Interventional endoscopy has evolved as an effective alternative to primary surgery for treating gastrointestinal perforation.

## 1 Introduction

Approximately 7% to 20% of cases of penetrating abdominal trauma involve gastric injury.^[1]^ Computed tomography (CT) is an important technique for identifying and diagnosing an abdominal stab wound. However, the use of only CT may lead to missed diagnosis. Gastroscopy, as an important supplementing technique, may not only allow identification of gastric injury, but may also facilitate prompt repair. A consensus has been reached that interventional endoscopy has evolved as an effective alternative to primary surgery for treating gastrointestinal perforation.^[2]^ Here, we report a case of blunt gastric perforation by a screwdriver that was successfully repaired by endoscopic clip closure.

## 2 Case report

The experimental protocol was established, according to the ethical guidelines of the Helsinki Declaration and was approved by the Human Ethics Committee of Capital Medical University Affiliated Beijing Friendship Hospital, China. Written informed consent was obtained from individual participant.

**Figure 1:**
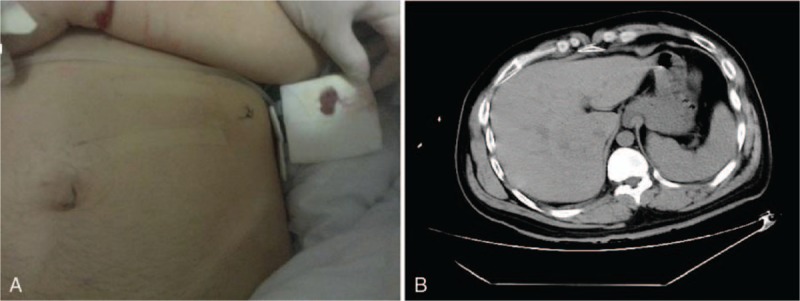
(A) Photograph of abdominal wound. (B) Computed tomography scan showing no evidence of abdominal wall perforation.

**Figure 2:**
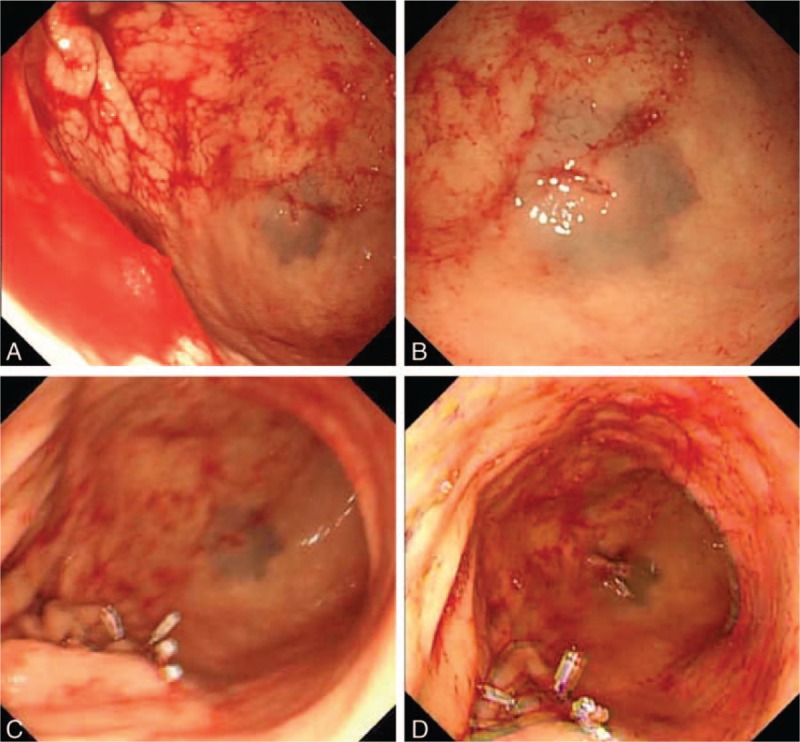
Endoscopic views of the wound and management. (A) Perforation and bleeding site in the greater curvature side. (B) Wound in the posterior gastric wall. (C) Endoscopic clip closure of the bleeding wound. (D) Endoscopic clip closure of the wound in the posterior gastric wall.

A 39-year-old male patient was admitted 3 hours after incurring an abdominal injury caused by penetration of a screwdriver. The attending doctor probed the injury and found a 1-cm wound, which did not penetrate the abdominal cavity. Local debridement and suturing were administered (Fig. 1A). During the following observation period, the patient repeatedly vomited large amounts fresh blood, totaling 3000 mL. His Blatchford score was 14, but there was no sign of peritonitis. Computed tomography (CT) was performed, and no evidence of gastric perforation was observed (Fig. 1B). Gastroscopic examination was conducted to determine the source of bleeding and showed projectile bleeding from a ruptured artery in the greater curvature side (Fig. 2A) and a 0.6-cm wound in the posterior gastric wall (Fig. 2B). Ten titanium clips were applied to stop the bleeding and close the wound (Fig. 2C and D).

**Figure 3:**
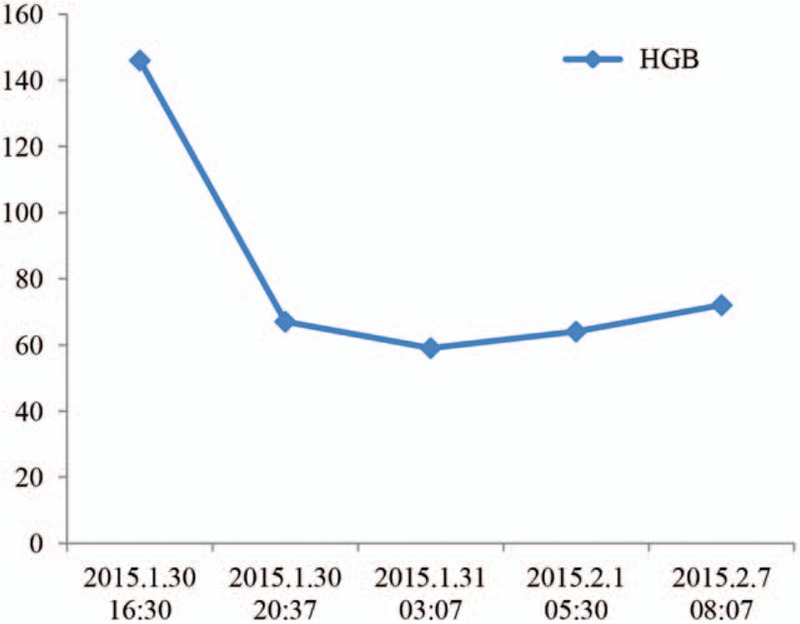
Change in the patient's hemoglobin level.

No more bleeding was observed after the operation, and therapies of volume expansion, acid suppression, hemostasis, and monitoring were applied. The patient received in infusion of 1000 mL red cells and 400 mL plasma, and his hemoglobin level was maintained at 7 to 9 g/dL over the observation period of 8 days (Fig. 3). No abdominal pain, upper gastrointestinal hemorrhage, or other complications were observed during the follow-up period.

## 3 Discussion

Gastrointestinal tract perforation can result from various causes, such as ulceration, inflammatory disease, a malignant tumor, iatrogenic factors, and so on.^[3]^ Obviously, it is necessary to identify the location and severity of a perforation correctly in order to determine appropriate management. A thorough medical history and physical examination are critical and provide important clues for further examination.^[4,5]^ CT scanning is a widely applied technique for the diagnosis of gastrointestinal injury with high sensitivity (94.2%) and positive-predictive value (98.8%). The effectiveness of CT scanning for identifying gastrointestinal perforation remains a subject of debate, with reported sensitivity rates varying from 76% to 93%.^[6]^ In the present case, gastric perforation was missed on physical examination and CT scanning in the initial diagnosis. A possible explanation might be that the gastric perforation caused by a screwdriver was similar to blunt dissection in surgery. After retraction of the screwdriver, the wound contracted and blocked the leakage of gastric contents temporarily. Therefore, no sign of gastric perforation was found on the physical examination or the CT scan. The gastric perforation was only later confirmed by gastroscopy for continuous hematemesis. Thus, it may be reasonable to suggest that gastroscopy be conducted routinely for patients with highly suspicious gastric injury, even if other examinations show no evidence of gastric perforation.

The main factors determining the selection of treatment for upper gastrointestinal perforations are the severity of complications and patients’ condition.^[2]^ Clavien-Dindo classification I and II perforations can be managed by conservative treatment, such as placement of a nasogastric tube, acid suppression therapy, and antibiotic therapy, whereas grade III perforations require endoscopic, surgical, or radiological intervention.^[7]^ Interventional endoscopy has become the first line therapy for gastrointestinal perforations in patients without sepsis.^[8,9]^ The mean success rate of interventional endoscopy for endoscope-related perforations has been reported to be as high as 90% (range: 70%–100%), and the success rates for postoperative perforations and chronic fistula are relatively lower (68% and 59%, respectively).^[2]^ Previously, Addley et al^[10]^ reported a successful case of a penetrating stab wound to the stomach treated by endoscopic clip closure, which was similar to the present case.

In summary, we suggest that gastroscopy should be conducted routinely for patients with a wound highly suspicious for gastric perforation, even if no sign of perforation is found on other examinations. In addition, interventional gastroscopy could serve as an effective technique for the management of gastric perforation.
